# Multi-organ benign and malignant tumors: recognizing Cowden syndrome: a case report and review of the literature

**DOI:** 10.1186/s13104-016-2195-z

**Published:** 2016-08-04

**Authors:** Maria Angela Gosein, Dylan Narinesingh, Cemonne Ann-Alicia Celeste Nixon, Sanjeeva Reddy Goli, Paramanand Maharaj, Alexander Sinanan

**Affiliations:** 1 Radiology Department, Port of Spain General Hospital, Port of Spain, Trinidad & Tobago; 2 Oncology, St. James Medical Complex, Port of Spain, Trinidad & Tobago; 3 Radiology, Medical Associates Hospital Limited, St Joseph, Trinidad & Tobago; 4 Radiology, University of the West Indies, St. Augustine, Trinidad & Tobago

**Keywords:** Cowden Syndrome, PTEN hamartoma, Radiology, Breast and endometrial cancer, Multiple meningiomas

## Abstract

**Background:**

Cowden syndrome is an autosomal dominant disorder with a predisposition to multiple benign and malignant tumors. In our patient, in addition to breast and endometrial malignancies as well as facial trichilemmomas, she was noted to have multiple meningiomas, pancreatic lipomas and lung cysts. These latter lesions have been noted in previous Cowden syndrome case reports, but are not included in the diagnostic criteria at this time. To our knowledge, this is the first case of multiple meningiomas in this syndrome. Further studies are therefore warranted to assess the significance of these findings in Cowden syndrome.

**Case presentation:**

A middle-aged Afro-Caribbean known endometrial carcinoma patient (post surgery and adjuvant radiotherapy), presented with a locally advanced breast carcinoma. She received neoadjuvant chemotherapy followed by a modified radical mastectomy and axillary lymph node clearance. Her past medical history included a sphenoid wing meningioma for which she received definitive external beam radiotherapy. She was also known to have a multinodular goiter, anal polyp and longstanding mucocutaneous lesions. Further workup revealed additional smaller meningiomas, a parotid arteriovenous malformation, a lung cyst and pancreatic lipomas. Overall, consortium criteria were met for the diagnosis of Cowden syndrome. Furthermore, genetic testing identified a pathogenic mutation in the PTEN gene. She will be closely followed with annual clinical examination, dermatologic assessment and screening colonoscopies. She will perform interval whole body contrast enhanced CT for continued surveillance for metastatic disease.

**Conclusion:**

Cowden syndrome is likely to be an under diagnosed condition, but critically important to identify due to its cancer predisposition. When encountering multi-organ tumors, diagnostic criteria for Cowden syndrome should be sought in order to increase the diagnostic rates. Cancer surveillance for carcinoma detection in the early and curative stages remains the most critical aspect of management.

## Background

Although rare, cowden syndrome (CS) or phosphate and tensin homolog (PTEN) hamartoma syndrome is an autosomal dominant disorder characterized by multiple hamartomas throughout the body with an increased lifetime risk of several carcinomas [1, 2]. These include breast, thyroid, endometrial, colorectal and renal carcinomas [2, 3]. Surveillance for early cancer detection is therefore essential to ensure optimal survival for patients afflicted with this syndrome. Due to its varied presentation however, this condition can be difficult to recognize and is often under diagnosed; although mucocutaneous features are present in 99 % of patients by the third decade of life [4]. Clinicians must therefore also be aware of the other manifestations of CS including macrocephaly, arteriovenous malformations, gastrointestinal polyps, testicular lipomatosis and thyroid nodules, in order to increase the diagnostic rates for this disorder [1, 2]. There is currently insufficient data to include meningiomas in the diagnostic criteria of CS due to its prevalence in the general population [2]. Meningiomas however have been described in a number of previous case reports [5] and the multiplicity of these lesions is of note in this patient. A thin walled lung cyst and pancreatic lipomas were additional findings in this patient’s imaging studies. These have been noted in previous case reports [6, 7] and possibly represent hamartomatous lesions. Further study is therefore warranted to ascertain the relevance of these findings to CS.

## Case presentation

A 57-year old Afro-Caribbean female presented with a T4N2M0 triple negative mucinous right breast carcinoma. She was 1-year post radical hysterectomy and adjuvant radiation for abnormal uterine bleeding, with the corresponding histology of endometrial carcinoma as well as multiple uterine leiomyomas. She had a 12-year history of left exophthalmos secondary to a sphenoid wing meningioma, for which she received external beam radiotherapy. She also had a history of rectal bleeding secondary to an anal polyp, which was resected and proved to be a tubular adenoma.

Several dermatological lesions were noted on examination, which the patient reported to be present ‘since childhood’. These included flesh colored papules in the perinasal region, warty papules on the tongue, hands and feet as well as palmar pits. A visible right neck swelling was also noted and further questioning revealed symptoms of dysphagia as well as a history of prior left thyroidectomy for a multinodular goiter. She did not report any shortness of breath, palpitations or significant weight loss. A left cheek swelling was palpated, which was pulsatile in nature. Our patient also had a thoracolumbar scoliosis and complained of symptoms of sciatica. Unfortunately, as she was estranged from her family, her family medical history and genetic testing of family members could not be obtained.

**Fig. 1:**
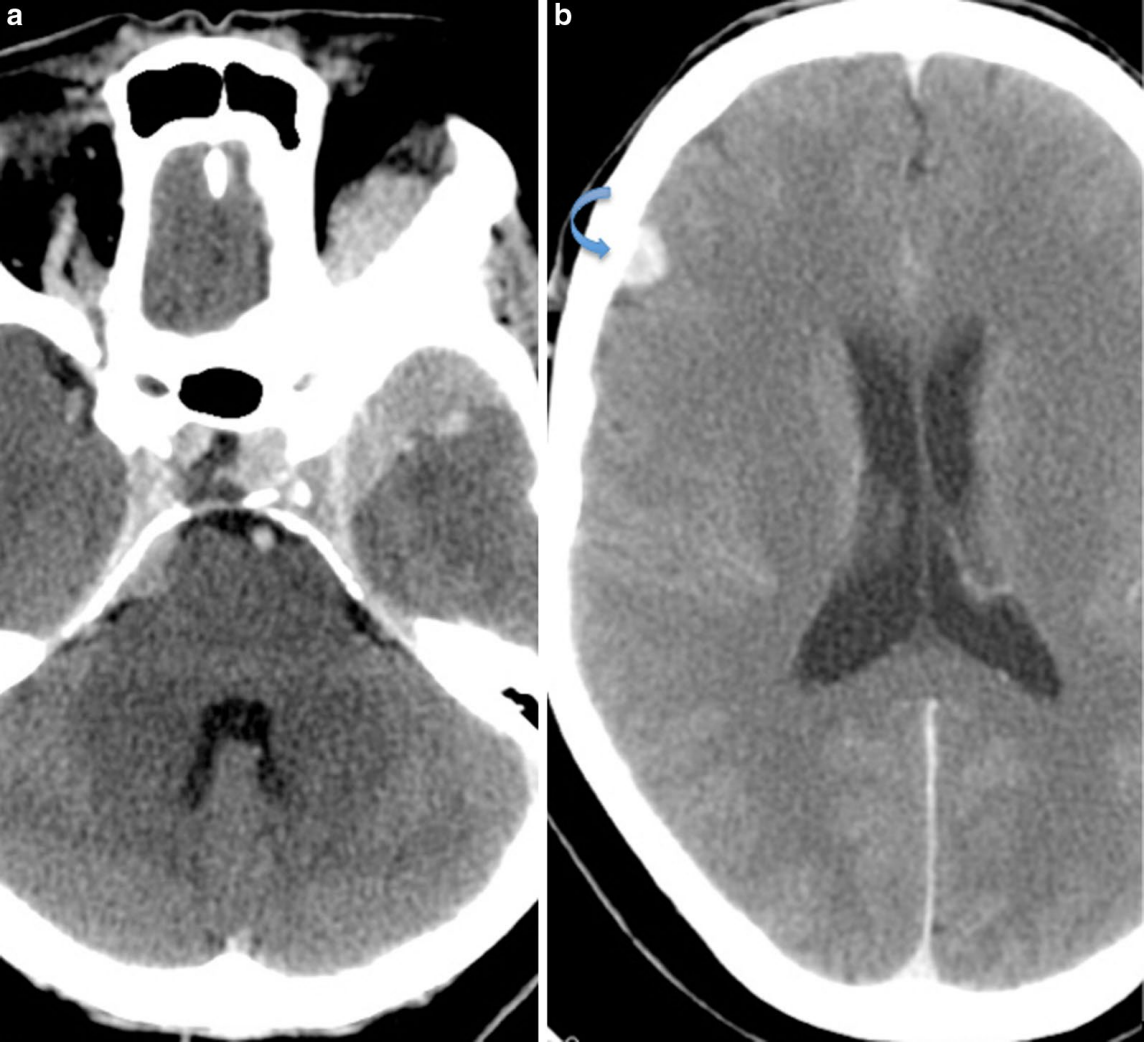
Axial contrast enhanced CT of the brain. **a** Large homogenously enhancing left sphenoid wing meningioma with associated hyperostosis and extension into the left anterior temporal fossa and left extraconal space. **b** Smaller well-defined enhancing extra-axial lesion in the right frontal lobe (*arrow*), consistent with a convexity meningioma

**Fig. 2:**
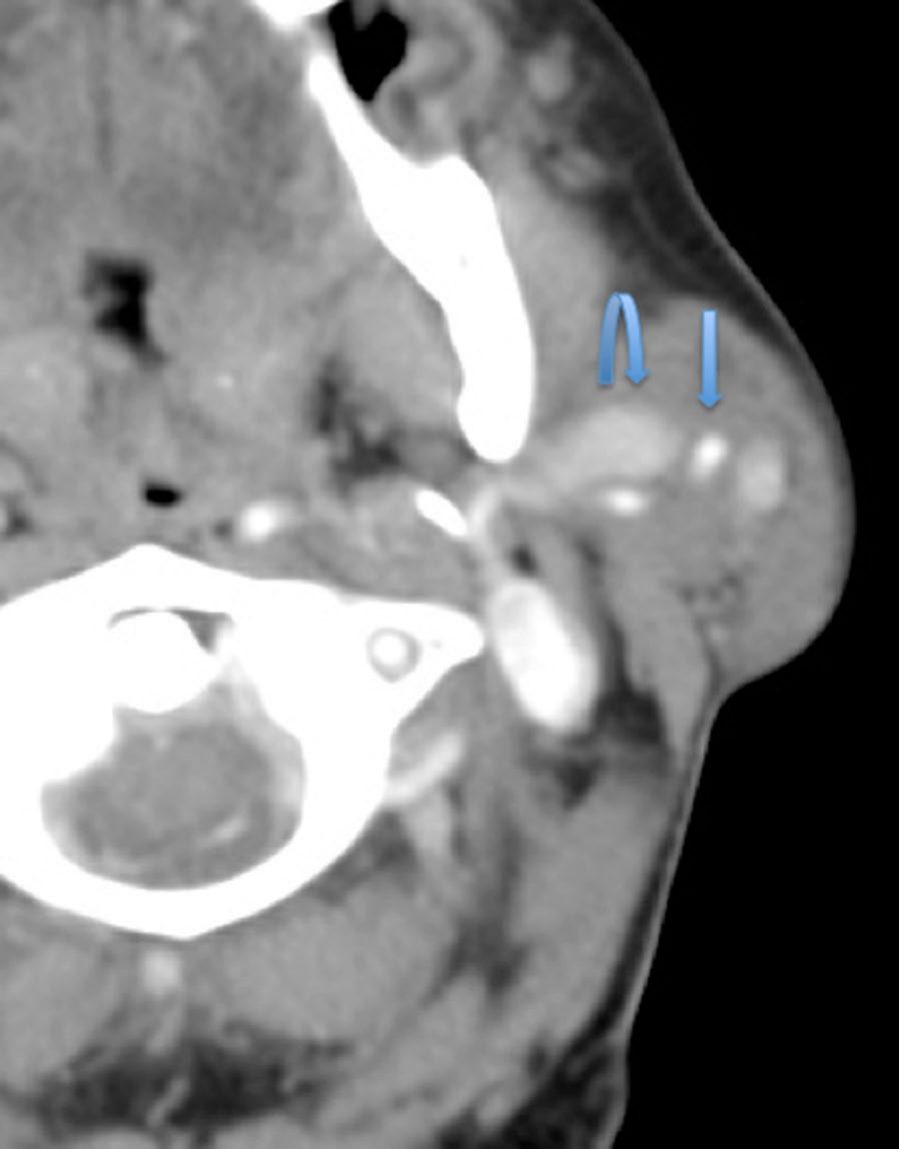
Axial contrast enhanced CT at the level of C1. Left parotid arteriovenous malformation with a left external carotid artery branch feeding vessel (*straight arrow*), along with an enlarged draining vein (*curved arrow*)

**Fig. 3:**
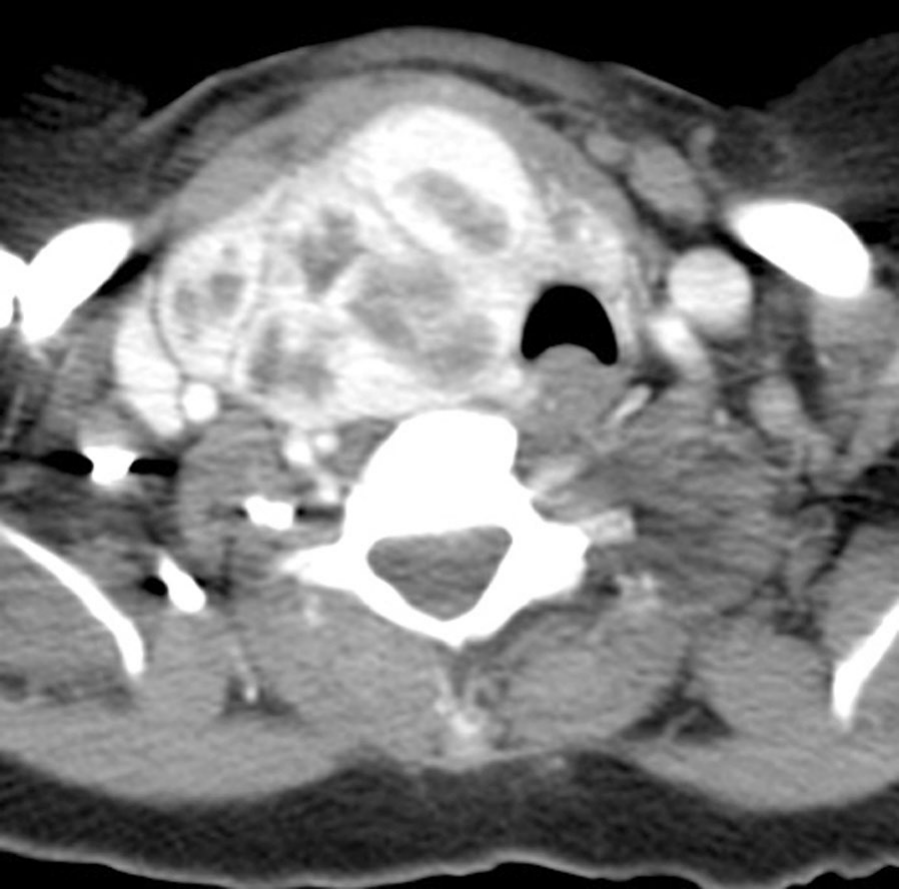
Axial contrast enhanced CT at the level of the thyroid gland. Left thyroidectomy noted with multinodular enlargement of the right thyroid lobe with marked tracheal deviation to the *left*

**Fig. 4:**
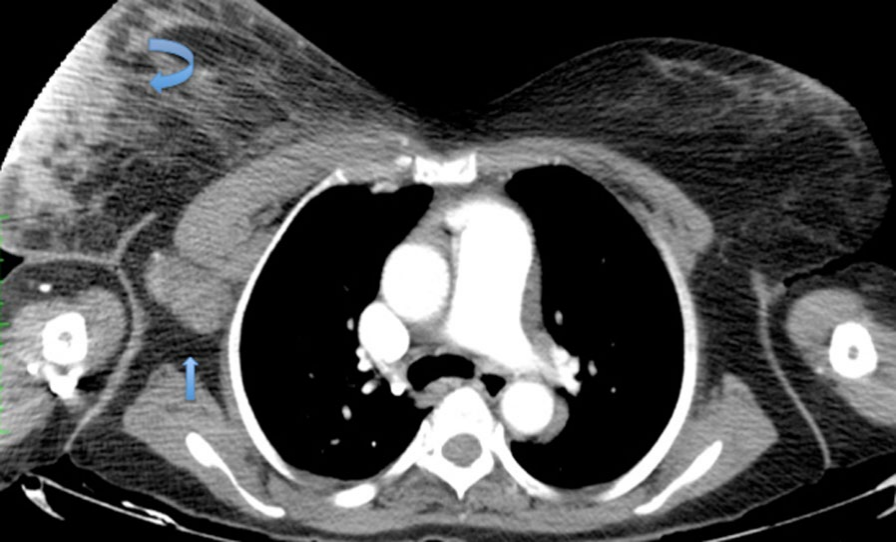
Axial contrast enhanced CT of the Chest. The right breast lesion was partially imaged (*curved arrow*) pre-operatively along with skin thickening and level I and II axillary adenopathy (*straight arrow*)

**Fig. 5:**
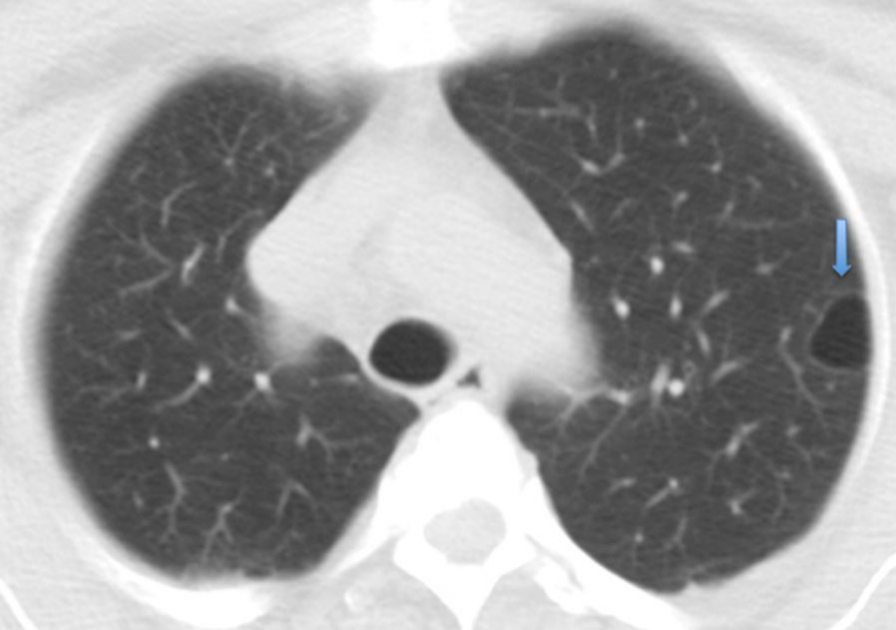
Axial CT of the Chest: Pulmonary window. A solitary thin walled lung cyst was present in the periphery of the left upper lobe (*arrow*)

Hematological and biochemical investigations as well as thyroid function tests were all within normal limits. Contrast enhanced CT of the brain, neck, chest, abdomen and pelvis was performed for staging purposes and to monitor known lesions. CT revealed a left sphenoid wing meningioma with extra-conal extension and resultant left optic nerve impingement and proptosis. Two other homogenously enhancing extra-axial lesions were noted, with one showing calcification, consistent with the presence of additional smaller meningiomas. These were stable on imaging for 5 years (Fig. 1). The pulsatile left cheek swelling corresponded with a left parotid arteriovenous malformation, with the arterial feeder arising from the left external carotid artery (Fig. 2). A left thyroidectomy was noted with multinodular enlargement of the right thyroid lobe and isthmus along with retrosternal extension and marked tracheal deviation to the left (Fig. 3). The right breast lesion was partially imaged on CT preoperatively, along with level I and II axillary adenopathy (Fig. 4). Of note was a thin walled lung cyst in the periphery of the left upper lobe (Fig. 5).

**Fig. 6:**
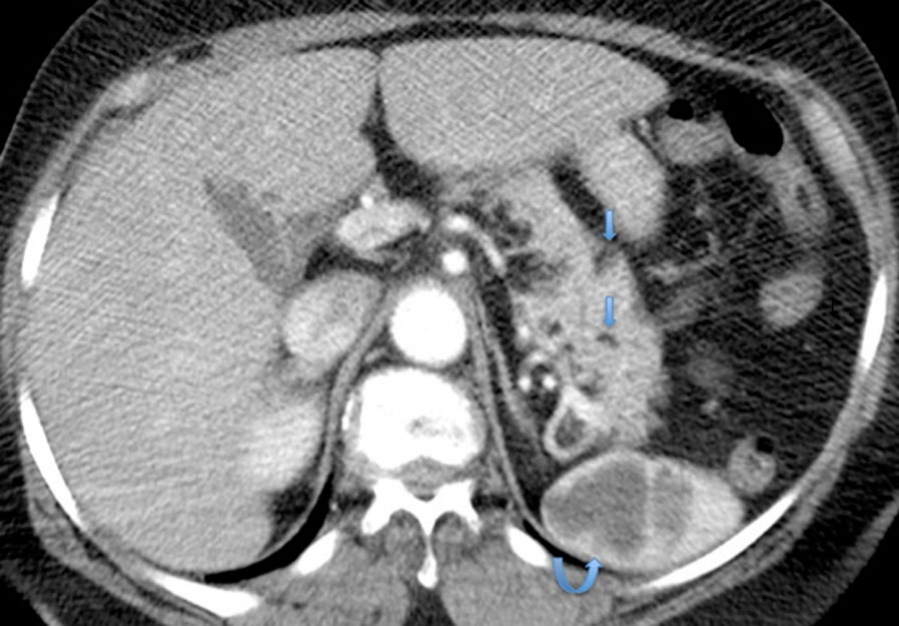
Axial contrast enhanced CT of the Abdomen. Multiple hypo enhancing splenic lesions were seen (*curved arrow*), likely representing hamartomas as well as subcentimeter fat density lesions within the pancreas (average HU = −70) (*straight arrows*)

Multiple hypo enhancing splenic lesions were seen, likely representing hamartomas, as well as several subcentimeter fat density lesions within the pancreas (Fig. 6). The pelvis showed post surgical changes with no evidence of local recurrence or lymphadenopathy. No metastatic disease was evident. Mammogram of the left breast showed no evidence of spiculated masses, clustered micro calcifications or architectural distortion. Our patient declined biopsy of her skin lesions however these were assessed by dermatologists and clinically confirmed to be facial trichilemmomas, acral keratosis, palmar pits and oral papillomas. MRI of the spine revealed a thoracolumbar scoliosis with spondylosis and multilevel disc disease however there was no evidence of metastatic disease, dural ectasia or extra medullary lesions. Genetic testing identified a pathogenic mutation in the PTEN gene: c.697C > T (pArg233*).

Following neoadjuvant chemotherapy, our patient underwent a right modified radical mastectomy with axillary lymph node dissection. Although she declined debulking operative intervention for the sphenoid wing meningioma, she was treated with external beam radiotherapy; with stability of this lesion noted post radiation. Prophylactic mastectomy for the contralateral breast was discussed at the multidisciplinary team meeting, however our patient opted to instead have regular clinical and imaging examinations for screening of the left breast.

There has been no clinical or radiological evidence of local or nodal recurrence of her endometrial carcinoma. FNAC of the right thyroid showed no evidence of malignancy, however the patient is considering thyroidectomy due to worsening symptoms of dysphagia. She continues to be monitored in the outpatient clinic setting for her sphenoid wing meningioma and parotid AVM, however she is not keen on operative management. For follow up, she will be offered annual thorough clinical examination including dermatologic assessment as well as screening colonoscopies. She will also perform interval whole body contrast enhanced CT for continued surveillance for metastatic disease. On this examination, her kidneys will also be closely monitored for renal cell carcinoma.

## Discussion

Cowden syndrome (CS) is an autosomal dominant disorder that is associated with the formation of hamartomatous tumors as well as a predisposition to a number of malignancies; mainly breast, thyroid and endometrial carcinomas [1, 2]. CS is estimated to occur in 1:200,000 patients however this is likely to be an underestimate due to current underdiagnosis [4]. CS is the prototype of the PTEN hamartoma tumor syndrome disorders and is linked to germline mutations in the phosphate and tensin homolog (PTEN) tumor suppressor gene [1, 2]. Molecular genetic testing can be performed to assess for this mutation however new prospective research identified these mutations in only 25 % of involved individuals [8]. The consensus diagnostic criteria should therefore be used as the mainstay of diagnosis at this time. This has recently been revised by Pilarski et al. [2] (Table 1).

**Table 1:** Phosphate and Tensin Homolog (PTEN) hamartoma tumor syndrome revised clinical diagnostic criteria [2]

*Major criteria*	
Breast cancer	
Endometrial cancer (epithelial)	
Thyroid cancer (follicular)	
Gastrointestinal hamartomas (including ganglioneuromas, but excluding hyperplastic polyps; ≥3)	
Lhermitte-Duclos disease (adult)	
Macrocephaly (≥97 percentile: 58 cm for females, 60 cm for males)	
Macular pigmentation of the glans penis	
Multiple mucocutaneous lesions (any of the following):	
Multiple trichilemmomas (≥3, at least one biopsy proven)	
Acral keratoses (≥3 palmoplantar keratotic pits and/or acral hyperkeratotic papules)	
Mucocutaneous neuromas (≥3)	
Oral papillomas (particularly on tongue and gingiva), multiple (≥3) OR biopsy proven OR dermatologist diagnosed	
*Minor criteria*	
Autism spectrum disorder	
Colon cancer	
Esophageal glycogenic acanthosis (≥3)	
Lipomas (≥3)	
Mental retardation (i.e., IQ ≤ 75)	
Renal cell carcinoma	
Testicular lipomatosis	
Thyroid cancer (papillary or follicular variant of papillary)	
Thyroid structural lesions (e.g., adenoma, multinodular goiter)	
Vascular anomalies (including multiple intracranial developmental venous anomalies)	
*Operational diagnosis in an individual (either of the following)*	
1. Three or more major criteria, but one must include macrocephaly, Lhermitte-Duclos disease, or gastrointestinal hamartomas; or	
2. Two major and three minor criteria	
*Operational diagnosis in a family where one individual meets revised PTEN hamartoma tumor syndrome clinical diagnostic criteria or has a PTEN mutation*	
1. Any two major criteria with or without minor criteria; or	
2. One major and two minor criteria; or	
3. Three minor criteria	

Women with CS were considered to have a lifetime risk of 25–50 % for developing breast cancer and 5–10 % for developing endometrial cancer, however more recent reports suggest an even greater risk of up to 85 % for breast cancer and 28 % for endometrial cancer [1–3]. Breast cancers may also develop in CS at an earlier age compared with the general population [2]. Anecdotally, benign breast and uterine disease such as fibroadenomas and leiomyomas have frequently been reported as clinical features of CS, however as these are common conditions in the healthy adult population, it is unclear whether the rate in CS is significantly increased [2].

Current data supports the hypothesis that both benign and malignant thyroid disease are a part of CS however again, due to the prevalence in the general population, this has a low predictive value for identifying those affected by mutations [2]. In particular however, follicular thyroid cancer appears overrepresented in PTEN mutation carriers compared with the general population [2]. Follicular thyroid cancer is thus included as a major criterion with papillary thyroid cancer and benign thyroid nodules considered as minor criteria [2] (Table 1). Other neoplasms including colorectal cancers, skin cancers and renal cell cancers are suspected to be associated with CS; with one study estimating the lifetime risk of colorectal cancer at 9 % and renal cell carcinoma at 34 % [2, 3].

Findings of an adult onset dysplastic gangliocytoma of the cerebellum or Lhermitte-Duclos disease (a benign, slow growing hamartoma) as well as macrocephaly are included as major criteria for the diagnosis of PTEN hamartoma tumor syndrome [2] (Table 1). Although a number of cases of meningioma have been reported in CS in the literature [5], there is insufficient data currently to confirm this association [2]. Multiple meningiomas were however noted in our patient on imaging. CT in our patient also demonstrated multiple lipomas in the pancreas as well as a thin walled cyst in the lung. These findings were noted radiologically in previous case reports [6, 7] with histological confirmation of a mesenchymal cystic hamartoma of the lung by Cottin et al. [7]. Future studies are therefore warranted to determine the significance of these imaging findings in the diagnosis of CS.

Trichilemmomas are lichenoid, skin-colored papules with a smooth surface. They are rarely sporadic and usually multiple at presentation of CS, in the central portion of the face [9]. The presence of at least 3 is thus included as a major criterion [2] (Table 1). They can however be mistaken for a common facial wart hence histopathology may be necessary. Other dermatological lesions include oral papillomas, mucocutaneous neuromas, acral keratosis and penile pigmentation [2]. As 99 % of individuals manifest with mucocutaneous stigmata by the 3rd decade of life, it is an extremely important heralding sign of the disease [4].

Most critical in the management of CS is surveillance for early cancer detection, resulting in improved overall survival. All CS patients should undergo an annual thyroid ultrasound scan and dermatologic evaluation. Women should receive an annual mammogram and breast MRI from age 30 along with annual transvaginal ultrasound and blind suction endometrial biopsies [1, 3]. Prophylactic mastectomy or prophylactic hysterectomy may also be considered after appropriate counseling. All adults with CS should have a colonoscopy beginning at age 35 as well as renal imaging every 2 years, beginning at age 40. These screening measures may also be undertaken 5–10 years before any known family history of a particular type of carcinoma (whichever is earlier) [10].

Interestingly, a number of familial syndromes have been linked by the shared loss of function of tumor regulator genes, leading to increased activity of the mammalian target of rapamycin (mTOR) pathway [11]. These syndromes include Neurofibromatosis type 1, tuberous sclerosis complex, Birt-Hogg-Dube, Peutz-Jeghers syndrome and Cowden syndrome [12]. This has led to clinical trials for mTOR inhibitors as a chemo preventative and therapeutic modality for these conditions [13, 14].

## Conclusion

Cowden syndrome is likely to be an underdiagnosed entity due to its diverse phenotypic features. When encountering multi-organ pathologies such as macrocephaly, arteriovenous malformations and benign breast and thyroid disease, CS should be considered with a search for other manifestations, particularly the characteristic trichilemmomas. Future studies are warranted to ascertain any significant links between meningiomas, pancreatic lipomas and lung cysts in the diagnostic criteria for CS. Although Cowden syndrome predisposes to a number of malignancies, screening measures can lead to early detection and improved survival and is therefore the hallmark of disease management at this time.

## Abbreviations

AVM: arteriovenous malformation
CS: cowden syndrome
CT: computed Tomography
FNAC: fine needle aspiration cytology
MRI: magnetic resonance imaging
mTOR: mammalian target of rapamycin
PTEN: phosphate and tensin homolog
